# Linear and Curvilinear Trajectories of Cortical Loss with Advancing Age and Disease Duration in Parkinson’s Disease

**DOI:** 10.14336/AD.2015.1110

**Published:** 2016-05-27

**Authors:** Daniel O. Claassen, David G. Dobolyi, David A. Isaacs, Olivia C. Roman, Joshua Herb, Scott A. Wylie, Joseph S. Neimat, Manus J. Donahue, Peter Hedera, David H. Zald, Bennett A. Landman, Aaron B. Bowman, Benoit M. Dawant, Swati Rane

**Affiliations:** ^1^Department of Neurology, Vanderbilt University, Nashville, TN 37235, USA; ^2^McIntire School of Commerce, University of Virginia, Charlottesville, VA 22904, USA; ^3^Department of Medicine, University of Virginia, Charlottesville, VA 22904, USA; ^4^Department of Neurosurgery, Vanderbilt University, Nashville, TN 37235, USA; ^5^Department of Radiology, Vanderbilt University, Nashville, TN 37235, USA; ^6^Department of Psychology, Vanderbilt University, Nashville, TN 37235, USA; ^7^Department of Biomedical Engineering, Vanderbilt University, Nashville, TN 37235, USA

**Keywords:** Parkinson’s disease, Cortex, MRI, Aging, Disease duration, Neurodegeneration

## Abstract

Advancing age and disease duration both contribute to cortical thinning in Parkinson’s disease (PD), but the pathological interactions between them are poorly described. This study aims to distinguish patterns of cortical decline determined by advancing age and disease duration in PD. A convenience cohort of 177 consecutive PD patients, identified at the Vanderbilt University Movement Disorders Clinic as part of a clinical evaluation for Deep Brain Stimulation (age: M= 62.0, SD 9.3), completed a standardized clinical assessment, along with structural brain Magnetic Resonance Imaging scan. Age and gender matched controls (n=53) were obtained from the Alzheimer Disease Neuroimaging Initiative and Progressive Parkinson’s Marker Initiative (age: M= 63.4, SD 12.2). Estimated changes in cortical thickness were modeled with advancing age, disease duration, and their interaction. The best-fitting model, linear or curvilinear (2^nd^, or 3^rd^ order natural spline), was defined using the minimum Akaike Information Criterion, and illustrated on a 3-dimensional brain. Three curvilinear patterns of cortical thinning were identified: early decline, late decline, and early-stable-late. In contrast to healthy controls, the best-fit model for age related changes in PD is curvilinear (early decline), particularly in frontal and precuneus regions. With advancing disease duration, a curvilinear model depicts accelerating decline in the occipital cortex. A significant interaction between advancing age and disease duration is evident in frontal, motor, and posterior parietal areas. Study results support the hypothesis that advancing age and disease duration differentially affect regional cortical thickness and display regional dependent linear and curvilinear patterns of thinning.

The distribution of cortical atrophy in Parkinson’s Disease (PD) is widespread, extending to frontal, temporal, posterior parietal and occipital regions [1]. Although the pathologic mechanisms of cortical atrophy are diverse, advancing disease pathology is characterized by alpha-synuclein deposition in these areas[2]. Based on the influential disease progression model presented by Braak and colleagues, early pathologic changes (stages 1-3) in PD are expressed in the brainstem, while later stages of disease (stages 4-6) are defined by the presence of Lewy Body and Lewy Neurite deposits in the cortex [3]. This late-stage deposition is thought to follow a ‘non-random’ pattern, beginning first in the allocortex (e.g. anterior olfactory nucleus) and progressing to higher order neocortical sensory and prefrontal regions (e.g. insular cortex and association cortex)[4]. While advancing Braak stage appears to correlate with broad cognitive symptoms [5], little data is available to clarify the relationship between duration of disease and age-related progression of cortical changes.

In normal aging, reductions in cortical thickness localize to the pre-frontal, temporal, and occipital cortices [6, 7]. However, whether PD-related changes in the rate and pattern of cortical atrophy are influenced by age is unclear [8, 9]. Teasing apart age-related from disease-duration relationships with pathophysiology or clinical progression is often difficult as they are both positively correlated with time. Indeed, age is one of the strongest risk factors for PD, atrophy is expected in the normal aging cortex[6], and advancing age is a risk factor for other ‘non-PD pathologic processes’ that may contribute to cortical atrophy (e.g. Alzheimer Disease pathology)[10]. Given apparent links between disease duration and increased motor and cognitive dysfunction, efforts to specify the underlying pattern and time course of cortical atrophy that account for patient age may have profound therapeutic impact and improve our understanding of the mechanisms underlying pathologic progression.

In this study, we aimed to distinguish the effects of disease duration from age related cortical atrophy in a large cohort of PD patients with varying disease duration and age. We determined linear as well as curvilinear relationships between disease duration and age with cortical thickness. We further compare these relationships to patterns of cortical thinning in normal aging. Our data allow an improved model of pathological progression in PD that accounts for age and disease duration across cortical structures.

## MATERIALS AND METHODS

### Participants

A cohort of 177 consecutive PD patients presented to Vanderbilt University between 2006 and 2013. All patients were diagnosed with idiopathic PD by a Neurologist trained in Movement Disorders, and met UK Brain Bank criteria for a diagnosis of PD. Patients were examined as part of a Deep Brain Stimulation (DBS) clinical evaluation protocol. Motor severity was defined using part III of the United Parkinson Disease Rating Scale (UPDRS) and performed by a single examiner: both in the withdrawn dopamine state (overnight withdrawal) and in the optional medicated state. In addition, patients and caregivers underwent detailed assessment defining the year of the first motor symptom. From this, disease duration was estimated based on the time from first symptom (or in rare cases when unclear, first described by a physician) to current assessment. An additional 53 healthy controls were selected from the publicly available databases; Progressive Parkinson’s Markers Initiative (PPMI; www.ppmi-info.org/data) and Alzheimer’s Disease Neuroimaging Initiative (ADNI; www.adni-info.org). Participant characteristics are described in Table 1. Age of assessment was similar between PD and older healthy controls. PD patients had mean disease duration of 10.3 years, with moderate severity as evidenced in the UPDRS III score On and Off medication. MR imaging parameters were similar to the ones obtained for PD patients. The Vanderbilt University Institutional Review Board approved the study, and all PD patients provided informed consent for the clinical protocol.

**Table 1:** Participant Demographics

	Parkinson’s Disease (n=177)	Healthy Controls (n=53)
**Gender (M:F)**	(121:56)	(39:14)
**Assessment Age (years)**	62.0 (9.3)	63.68 (12.23)
**Disease Duration**	10.3 (4.8)	—
**UPDRS III Off**	40.8 (12.9)	—
**UPDRS III On**	18.7 (10.0)	—

Values are presented as mean (s.d.); UPDRS III On = Patient evaluated in optimal dopamine state; UPDRS III Off = Patient evaluated 12-16 hours following dopamine withdrawal. M = male; F = female; UPDRS = Unified Parkinson’s disease rating scale

For up-to-date information on the PPMI, visit www.ppmi-info.org. *Principal Investigator Michael W. Weiner leads ADNI, which was launched in 2003.* ADNI tests whether serial MRI, positron emission tomography (PET), biological markers, and clinical and neuropsychological assessment can be combined to measure the progression of mild cognitive impairment (MCI) and early Alzheimer’s disease (AD).

### Neuropsychological Status

All patients completed baseline neuropsychological testing, which preceded the MRI by at most 3 months. Neuropsychological testing was recorded as scaled scores (based on participant age and education), ranging from 1-20, with a score if 10 being the 50 percentile, and S.D. of 3. We report mean and (S.D) of these scores in Table 2, emphasizing that patients performed lower in measures of executive function (Trails A and B), letter fluency, and memory encoding (Word List 1). These results provide additional cognitive characterization of this cohort, emphasizing that patients exhibited a cognitive profile consistent with moderate PD.

**Table 2:** Cognitive Profile of PD patients

Cognitive Assessment	Number tested	Mean (S.D) Scaled Score
Dementia Rating Scale^[33]^	153	12.0 (2.5)
Trails A^[34,35]^	177	7.6 (3.0)
Trails B^[34,35]^	177	8.1 (3.0)
Judgment of Line Orientation^[36]^	177	10.7 (2.9)
Letter Fluency^[37]^	177	9.4 (2.5)
Word List 1^[38]^	177	8.8 (3.4)
Word List 2^[38]^	177	11.3 (2.5)

Note: Trails A/B, and Judgment of Line Orientation scores were calculated using Mayo’s Older Americans Normative Studies (MOANS) norms.

### MRI acquisition

All PD patients underwent a T1-weighted anatomical MRI (3-Tesla Philips Achieva scanner [Philips Medical Systems, Best, The Netherlands] with eight-channel SENSE coil reception). General sedation was performed for reduced discomfort due to placement of bone markers. Brain imaging was obtained using a 3D turbo field echo acquisition with a spatial resolution = 1 × 1 × 1 mm^3^, resolution = 256 × 256, slices = 170, TR/TE = 7.92/3.65 ms. For controls, the acquisition parameters were: spatial resolution = 1 × 1 × 1 mm^3^, 0.9 × 0.9 × 1.2 mm^3^, 1 × 1 × 1 mm^3^, resolution = 256 × 256, TR/TE = 6.87-.92/2.98-3.15 ms.

### Cortical Thickness Measurement

A standard FreeSurfer pipeline was applied to all the T1-weighted MRI images to intensity normalize, skull-strip, and segment brain tissue. The choice of imaging parameters closely matched the FreeSurfer recommended parameters. Han et al. describe that segmentation inaccuracies are minimal with FreeSurfer recommended imaging parameters and do not affect study outcome, thus reducing the need to verify segmentation accuracy for each subject [11]. Each subject’s data was registered to the standard FreeSurfer brain (fsaverage). The Desikan-Killany atlas was used to identify cortical brain regions and evaluate cortical thickness per region. Previous studies have shown that acquisition types and resolution differences do not affect the robust cortical thickness measurements conducted using FreeSurfer [11-13].

### Modeling cortical thickness over advancing age and disease duration

The primary purpose of this analysis was to model the trajectory of cortical decline and determine if the pattern of decline followed a linear or curvilinear pattern. We modeled cortical thickness over advancing age, disease duration, and subsequently assessed the combined effect of age and disease duration on cortical thickness. Statistical analysis was performed using R software[14]. The best-fit model conforming to a linear or curvilinear (2^nd^ or 3^rd^ order natural splines) relationship was defined using the minimum Akaike Information Criterion (AIC)[15]. Based on the model results, we determined if the fit was linear, and in cases where it was curvilinear, categorized the model based on the trajectory of the best fit. We illustrated these rate-models onto 3-dimensional (3-D) cortical maps, using regions defined in the Desikan-Killany Atlas[16], thus visualizing linear and curvilinear relationships between cortical thickness and advancing age and disease duration on a 3-D brain. Since our study was cross-sectional in nature and relied on patients who presented for DBS assessment, one limitation was that fewer subjects (n=17) had disease duration <5 years, thus the confidence intervals on these estimates with minimal observations are large, and falsely illustrates a trend of increasing cortical thickness with disease duration. These patients were part of an early PD stimulation trial [17]. These patients were earlier in the disease course, and motor symptoms were not as severe as one would encounter in a routine DBS population. Despite this, our sample size was large enough to provide reliable estimates as to the nature of cortical atrophy over age and disease duration.

## RESULTS

**Figure 1.**
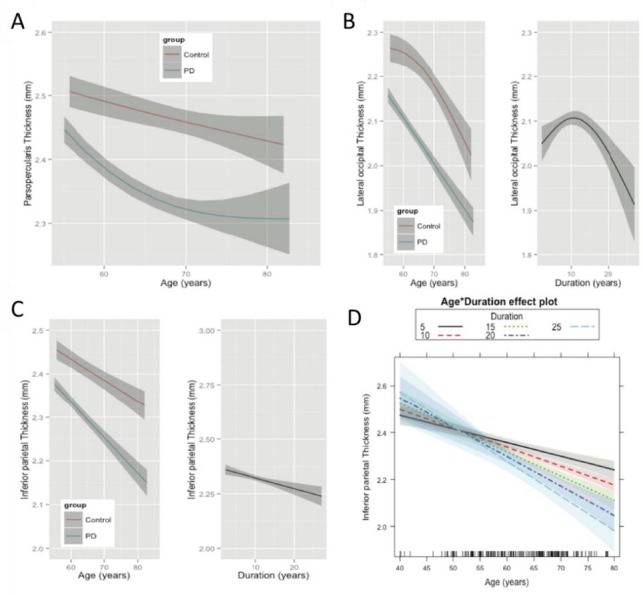
Models of age and disease duration effects on cortical thickness **A**) Age effects in the pars opercularis are different in PD (blue) compared to healthy controls (red). PD patients show a non-linear trajectory (blue) of ‘early decline’, which stabilizes. Rate of cortical thinning with age in PD is different in this frontal region, likely due to disease processes. However, cortical atrophy rate with respect to increasing disease durations was not significant. **B**) Age effects in the lateral occipital cortex conform to preservation of the cortex in the early years and an accelerated late decline in healthy controls (late decline), while in PD gray matter atrophies continuously with age (left panel). PD patients also showed a significant effect of disease duration alone, independent of age (right panel). **C**) Unlike Figure 1a, the parietal cortex shows a linear rate of cortical thinning with age in both controls and PD. However, cortical thinning in PD appears to be faster than in controls (left panel). Cortical thickness is also linearly dependent on duration of PD. Furthermore, increasing disease duration significantly increases the rate of cortical thinning (right panel). **D**) The interactions between age and disease duration in PD. Older patients with longer disease duration have a greater linear rate of cortical loss in the inferior parietal cortex.

We identified both linear and curvilinear patterns of cortical thinning over time by either age or duration of disease. Curvilinear patterns are best described as either (1) “early decline” (where atrophy occurs early on, then stabilizes), (2) “late decline” (stable cortical thickness followed by accelerating thinning), or (3) “early-stable-late” (this pattern conforms to early loss, stable thickness, then ensuing accelerated loss). Illustrative patterns of these different models of cortical changes are emphasized in Figure 1, where we show representative fits for cortical thickness in older healthy controls and in PD patients. For example, a differential influence of age on the pars opercularis was observed between PD and healthy controls (Figure 1a). In this cortical region a linear rate of cortical loss with aging is evident in healthy controls, while PD patients are best fit by a steep rate of cortical thinning early on that stabilizes with advancing age (early decline). In other cortical regions, disease duration and age effects show distinct trajectories in PD. For example, cortical thinning in the lateral occipital cortex accelerates with advancing age in healthy adults (late decline), while in PD the effect of age follows a steep linear rate (Figure 1b). With advancing disease duration, a rapid rate of cortical thinning occurs when disease duration has advanced beyond a decade (late decline). Furthermore, the linear rates of cortical loss differ between healthy controls and PD patients. Figure 1c illustrates one such example, wherein the rate of thinning in the parietal cortex is more pronounced in PD patients than in controls. Of note, this same region displays an interaction between age and disease duration in which the linear rate of cortical thinning over age increases with disease duration (Figure 1d), suggesting older patients with longer disease duration suffer a greater linear rate of cortical loss.

**Figure 2.**
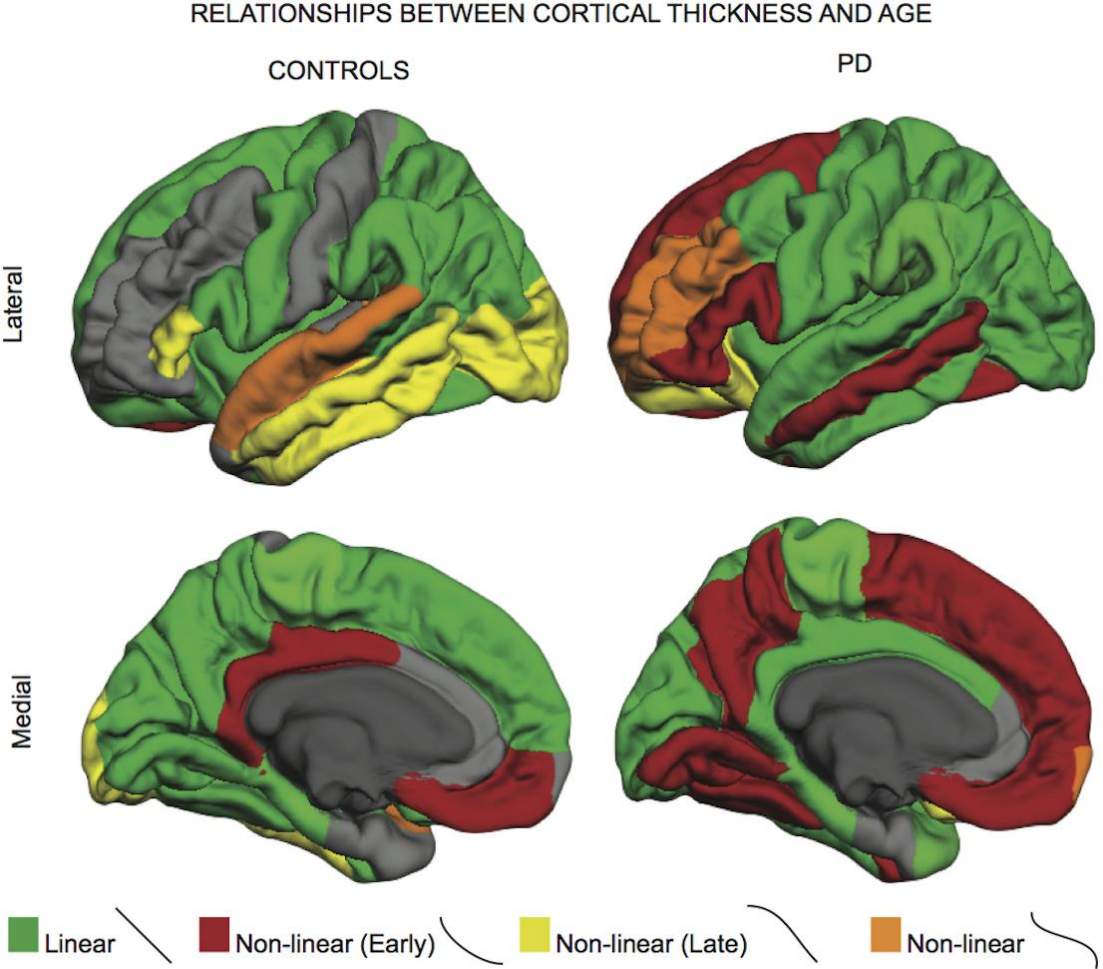
The effect of age on cortical thickness in healthy Ccontrols and Parkinson’s disease Green highlighted regions represent those regions that follow a linear rate of atrophy. Red highlighted regions depict rates of early decline (decreasing quickly at first, then stabilizing), while regions in yellow depict regions that atrophy faster over time (stable at first, then decreasing quickly). Regions in orange depict early decline, stabilization, followed by late decline, which is notable in later decades of life (early-stable-late). PD patients show characteristic frontal cortical thinning with age.

To aid in the conceptualization of these complicated modeling relationships, we illustrate the differing rates of cortical changes on a 3-D brain. Figure 2 illustrates cortical subregions (as defined by the Desikan-Killany Atlas) with a significant (p<0.001) relationship between cortical thinning and age in either healthy controls or PD patients. Notable differences between controls and PD patients exist in the frontal, cingulate and precuneal regions. The frontal regions are most distinct from controls, where curvilinear trajectories (especially early decline) are more common in prefrontal, medial frontal, and dorsolateral prefrontal regions. Overall, PD patients appear to suffer from early age-related cortical thinning, but the nature of this relationship with disease duration is further clarified when considering disease duration and age relationships.

**Figure 3.**
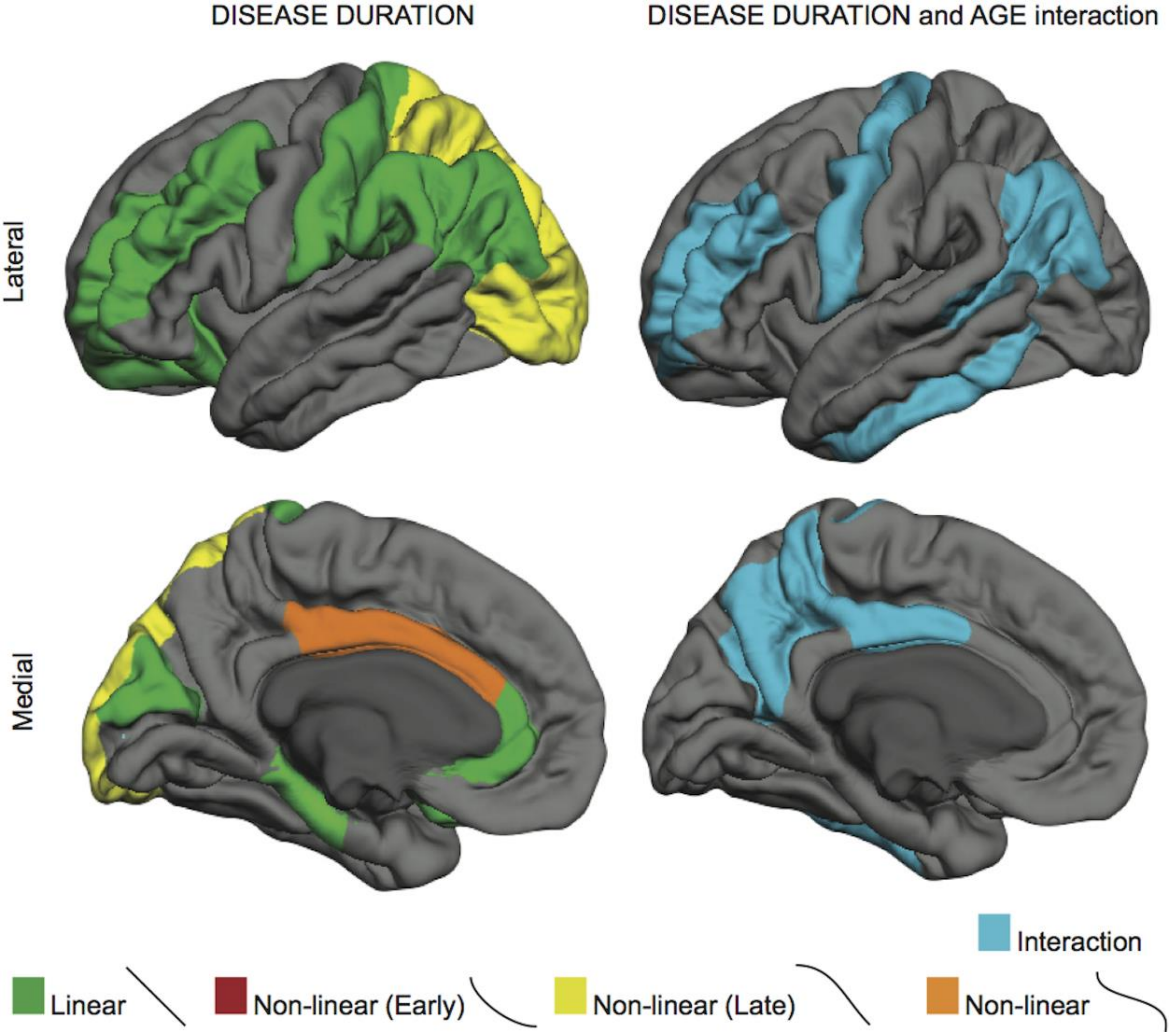
The effects of disease duration, and its interaction with age, on cortical thickness in Parkinson’s disease Regions in yellow depict regions that atrophy faster over time (concave down, decreasing curve or late decline). Regions in blue shown in the right column are regions showing an interaction between age and disease.

Cortical subregions showing a significant (p<0.001) influence of disease duration, or a disease-duration by age interaction effect, on cortical thickness are illustrated in Figure 3. While frontal and parietal regions show linear rates of cortical thinning, occipital subregions conform to a late decline, accelerating with advancing disease duration. Occipital cortical changes that occur with advancing disease duration do not appear to be a result of aging, as the best model for posterior regions with age in PD was a simple linear relationship. These results suggest that cortical pathology in the occipital region occurs with advancing disease duration (in our model, greater than 10 years of disease duration). Additionally, regions depicted in blue show significant interaction between age and disease duration. Here we see that key frontal, motor, and parietal regions are especially susceptible to cortical loss in older patients with longer years of disease duration.

## DISCUSSION

This study provides novel insights into the nature of cortical changes over the course of PD. Increasingly, quantitative imaging tools are being used to link disease-related symptoms to regional cortical thickness differences, yet there remains an incomplete understanding of the location and trajectory of cortical atrophy with advancing age and disease duration in PD. This work demonstrates that the nature of the relationship between increasing age and reduced cortical thickness differs between PD patients and healthy controls. While age-related cortical thinning is an established phenomenon, our observation that this age-effect is influenced by disease in a cortical sub-region dependent manner suggests a more complex relationship between age and PD than previously appreciated. A curvilinear trajectory of cortical thinning with age, where decline occurs early on (in our cohort from 50 to mid 60s), is regionally most evident in the frontal cortex and precuneus. Since age and disease duration are inherently correlated, one possible explanation for this finding is that younger patients are more prone to cortical changes as a consequence of early PD-related pathology. In an attempt to separate the effects of aging and disease duration, we compared the best-fitting models of changes in cortical thickness for disease duration versus aging. Our results show that cortical thickness over increasing disease duration follows a different trajectory to that of aging. Specifically, increasing disease duration is associated with a linear trajectory of thinning localized to the frontal and parietal regions, and a curvilinear pattern (accelerating decline after a decade of disease) to the occipital cortex. Finally, we show that frontal, parietal and temporal cortical regions appear synergistically susceptible to both increasing age and disease duration, emphasizing the empirical observations of neurologists that the effects of PD may worsen with age. Our findings are also consistent with longitudinal studies showing that with advancing age PD patients are more likely to experience progressive cognitive symptoms, balance problems, and reduced motor response to medications. For instance, clinical symptoms of gait dysfunction and cognitive impairment are associated with more widespread cortical dysfunction, and emerge later in life with an advanced disease course [18-22].

### Age effects on cortical thickness

Our best-fit models in healthy controls match previously reported age effects on cortical thinning. Cortical areas most susceptible to atrophy with age include the superior parietal, and the superior and middle frontal regions [23]. Slower rates of cortical thinning are notable in the dorsolateral prefrontal, temporal, and motor cortices with advancing age [6, 23]. Some regions are unaffected with age, while the trajectory of cortical thinning can appear linear, and also accelerate (particularly the occipital lobe) with older age [7]. Age-related cortical decline is attributed largely to a variety of non-pathological processes including reduced dendritic arborization and synaptic density[24]. In PD, age related cortical changes may also be attributed to a wide range of pathologic processes including reduced synaptic input from projection fibers or cell death associated with alpha-synuclein accumulation. In our PD cohort, the entire cortex appears susceptible to age related atrophy, but frontal, middle temporal, and cuneus/precuneus follow a curvilinear pattern. Most of these regions, especially frontal and precuneus, change earlier than observed in healthy controls. Previous studies have reported that PD patients with longer disease duration, cognitive impairment, or dementia have reduced cortical thickness or volume in these same regions [25-27], supporting the clinical relevance of our observations. Critically, our results suggest that the trajectory of decline in PD-aging is greater than those seen with non-PD aging, thus implicating a PD-specific pathologic process.

One possible explanation for the curvilinear (early decline) trajectory in these areas is that posterior cortical structures, such as the cuneus and occipital lobes, receive dense cholinergic input from the nucleus basalis of Meynert [28]. Early monoaminergic and cholinergic denervation may account for early age related atrophy in these areas [29]. Furthermore, in PD, increased neocortical amyloid deposition is associated with advancing symptoms such as gait instability and freezing[30], and accumulating amyloid deposition with advanced age may explain why we see a steeper rate of parietal thinning with aging in PD. Broadly, what accounts for the dramatically different trajectories of cortical atrophy with aging in PD is likely a combination of diverse pathophysiologic mechanisms, but the similar topography to that presented in the Braak model suggests that these changes may ultimately be an additive result of accumulating cortical PD-related pathogenic processes. Future studies are needed to address what accounts for dissimilar aging process in PD.

### Timing and curvilinear progression of cortical atrophy in Parkinson’s disease

The patterns of regional cortical thinning suggest that the neuropathological processes in PD act differentially across cortical regions and timescales. While it is possible that this simply reflects regional-specific vulnerabilities to the same pathological process, it seems more likely to reflect vulnerabilities to different aspects of advancing neuropathology, where subregion-dependent rates of thinning correspond to the emergence of distinct pathologic features over time. Later stages of the Braak model (stage V-VI) emphasize progressive alpha-synuclein accumulation in the sensory association areas—especially the middle temporal and visual association cortices which are linked to visual hallucinations [31]. We see diffuse cortical thinning, with predilection to the higher order sensory association cortices with longer duration of PD symptoms. It appears that frontal, middle temporal, and occipital regions exhibit more pronounced thinning with increasing disease duration [27]. While frontal and parietal regions decline linearly with disease duration, the occipital cortex thinning appears to dramatically accelerate with increasing disease duration [27]. Clinically, this mirrors prior work describing early cognitive changes in PD that arise from executive control deficits linked to reduced dopaminergic modulation of frontal-striatal circuitries, followed by a more ‘posterior’ cortical syndrome (characterized by impairments to visual-spatial and memory processes) later in disease [32]. These findings thus support empirical clinical evidence emphasizing that the nature of cortical pathology in PD begins anteriorly and advances posteriorly.

### Study implications and limitations

This cross-sectional study provides compelling evidence that PD is associated with curvilinear rates of cortical atrophy, independent from what would be expected by increasing age alone. The regional and temporal advancement of cortical atrophy is consistent with the progression described by Braak and colleagues, where the pathologic findings are first evident in the allocortex and progress neocortically. Curvilinear rates of atrophy suggest that distinct regions are susceptible to pathologic changes at different stages of PD. The cause of cortical thinning in PD is not clear, but alpha-synuclein related pathology likely contributes. Our data may also suggest that future studies of cortical differences between PD populations of interest should account for disease duration and not merely age, as advancing disease duration is associated with a significantly different trajectory of regional cortical atrophy. Not accounting for disease duration will emphasize PD-related changes that occur with age while potentially ignoring the desired disease symptom associations [26]. Additionally, linear rates of atrophy may inadequately capture the trajectory of PD-related cortical changes [25]. This modeling approach can guide future studies addressing longitudinal cortical changes in PD, by emphasizing susceptible regions and putative trajectories of change.

We acknowledge several important limitations from this study design. First, this is a cross-sectional study that warrants validation with longitudinal data. Second, this convenience cohort included patients who presented for consideration of DBS. These patients typically suffer from increased motor complications, and may not reflect the complete range of idiopathic PD. However, an advantage of this cohort is that all patients had longitudinal evaluations by a Movement Disorders neurologist and substantial improvements on dopamine therapy, thus reducing the likelihood of inclusions of patients with non-PD pathology. Notably, the cohort had fewer subjects (n=17) with disease duration <5 years. Despite these limitations, our findings show that PD is associated with a different age-related change in cortical thickness compared to healthy controls, and the trajectory of cortical thinning with disease duration follows previously reported autopsy and clinical phenomenology. Emphasis on improved quantitative staging of PD will improve clinical models of disease severity, and this staging may benefit from inclusion of both clinical and radiologic data. In summary, our results appear consistent with clinical findings that older PD patients with longer disease duration have a different disease trajectory. Advancing age and disease duration are key risk factors for dementia, where visual-spatial, memory, and executive dysfunction typify the cognitive course of PD. Results emphasize the need for non-linear models of disease, which will offer added insights to the pathophysiologic PD process.
